# The evolution of extraordinary self-sacrifice

**DOI:** 10.1038/s41598-021-04192-w

**Published:** 2022-01-07

**Authors:** D. B. Krupp, Wes Maciejewski

**Affiliations:** 1 Department of Interdisciplinary Studies, Lakehead University, Orillia, ON Canada; 2 Department of Mathematics, San José State University, San José, CA USA

**Keywords:** Evolutionary theory, Social evolution

## Abstract

From a theoretical perspective, individuals are expected to sacrifice their welfare only when the benefits outweigh the costs. In nature, however, the costs of altruism and spite can be extreme, as in cases of irreversible sterility and self-destructive weaponry. Here we show that “extraordinary” self-sacrifice—in which actors pay costs that exceed the benefits they give or the costs they impose on recipients—can evolve in structured populations, where social actions bring secondary benefits to neighboring kin. When given information about dispersal, sedentary actors evolve extraordinary altruism towards dispersing kin. Likewise, when given information about dispersal and kinship, sedentary actors evolve extraordinary spite towards sedentary nonkin. Our results can thus be summed up by a simple rule: extraordinary self-sacrifice evolves when the actor’s neighbors are close kin and the recipient’s neighbors are not.

## Introduction

From an evolutionary perspective, self-sacrifice takes two broad forms. *Altruism* reduces the lifetime fitness of the actor and increases that of the recipients. Conversely, *spite* reduces the lifetime fitness of both the actor and the recipients^1–3^. As Hamilton demonstrated with his inclusive fitness theory^4,5^, both can emerge if the recipients are more (in the case of altruism) or less (in the case of spite) likely than average to carry identical copies of the focal allele causing the behavior. Genetic associations like these typically arise as a result of common ancestry, because genealogical kin are more likely than nonkin to bear identical copies of one another’s alleles. Thus, consanguinity allows the actor to trade some fraction of its own reproduction for that of the recipients^6^.

A half century after Hamilton’s seminal publications, we have learned a great deal about the evolution of self-sacrifice^7^. Nevertheless, there remain problems that have eluded rigorous analysis. Here we focus on one such problem: the evolution of *extraordinary self-sacrifice*, whereby an actor pays a net cost $C > 0$ that exceeds the net benefit *B* they give to, or the cost they impose on, the recipients—that is, summed across all recipients, $C > |B|$. Such behaviors present a challenge since the actor does not stand to gain from the interaction, even over the long run, and since the powers of genetic association are often constrained. In part, this is because recipients are never more likely than the actor to bear copies of the actor’s alleles. And, in part, this is because the reproductive effects of the “primary” interaction between the actor and recipients can also lead to “secondary” effects that ripple through the population, affecting the actor’s and recipients’ neighbors. If those neighbors are kin, then the secondary effects lead to competition that can mitigate the strength and direction of selection^5,6,8–14^.

The combination of these two forces—bounded consanguinity and competition among kin—has led to a widespread, if tacit, assumption that the reproductive exchange between actor and recipients can only work when the total cost to the actor does not exceed the total benefit to the recipients, setting a theoretical limit on the evolution of self-sacrifice of $C < |B|$ (e.g. [4, 5, 15–18]). All else being equal, then, individuals are expected to value their own reproduction more than the reproduction of others. Accordingly, it remains difficult to explain the existence of adaptations that exact severe costs on actor fitness, such as the division of labor between somatic and germ cells or the irreversible sterility and self-destructive weaponry found in numerous eusocial insect species^19,20^, without resorting to the assumption that the consequences to the recipients must outweigh the cost to the actor.

While we do not deny that this assumption may hold over a wide range of cases, we show that, under certain conditions, it can be relaxed—allowing extraordinary self-sacrifice to evolve. Our argument fits entirely within an inclusive fitness framework (extending [﻿^9^, ^10^, ^13^, ^21^–^23^]), and rests on the ability of actors to steer costs away from neighboring kin and benefits towards them. The inclusive fitness approach is intuitive because it reflects the causal relationships among the affected parties: a focal allele causes an actor to perform an action that affects its own fitness as well as that of the recipients, and these effects in turn affect the fitness of others in the population, such as the actor’s and recipient’s neighbors^24,25^. The inclusive fitness *effect*, denoted $\Delta {W}$, is the sum of each of these individual fitness effects, weighted by the probability that the affected parties bear copies of the focal allele identical by descent. Though we acknowledge the controversy associated with inclusive fitness^7,26^, we have chosen this approach as it produces here a clear, productive, and explanatory decomposition of the effects of self-sacrifice within a population. Of course, this does not preclude other approaches that may reveal similar results, as we take up in the Discussion.

## A perspective from Hamilton’s rule

Consider, generally, a homogeneous population large enough that stochastic variation in population size can be ignored, and allow a genetically encoded behavior to emerge in interactions between pairs of individuals. This behavior causes the actor to reduce its fitness by a total amount $C > 0$ in order to increase or decrease the recipient’s fitness by a total amount *B*. Denoting a measure of genetic similarity—which can have different interpretations and mathematical formulations, made precise below—between the actor and recipient with *R*, we have the classical form of Hamilton’s Rule^27^:

$$\begin{aligned} RB > C. \end{aligned}$$

If we interpret *R* as the probability *Q* that the actor and recipient carry identical copies of the allele encoding the behavior (equivalent to an “others-only” coefficient of consanguinity in haploid populations^22,28^), as is commonly done, then the limits of $0 \le R \le 1$ imply that an extraordinarily costly social behavior—one for which $C > |B|$—cannot emerge in the population.

A common expansion of the definition of *R* involves normalizing the coefficient of consanguinity between the actor and the recipient by the coefficient of consanguinity between two different, random members of the population^8^, denoted $\bar{Q}$:

$$\begin{aligned} R = \frac{Q - \bar{Q}}{1 - \bar{Q}}. \end{aligned}$$

This form of relatedness captures the genetic similarity of individuals relative to a population mean genotypic value. Whereas this expression may seem to increase the explanatory scope of Hamilton’s Rule, the population mean $\bar{Q} \approx 0$ in very large populations, and so *R* simply reduces to *Q*. Accordingly, the limits of $0 \le R \le 1$ still apply, precluding the evolution of extraordinary self-sacrifice.

The picture begins to change, however, once we identify the effects of potentially different classes of actors and of population structure. In the initial formulation of Hamilton’s Rule, no constraints were placed on the size of the population—only that it be “big enough.” If we further make the reasonable assumption that the relative contributions of individuals to future generations in a corresponding neutral process are held constant—that is, that reproductive value is conserved^29^—then any net increase in the fitness of one party must be offset by a corresponding net decrease in the fitness of another party; an assumption of population inelasticity. Moreover, if the population is organized into subpopulations, then these secondary effects, denoted $\hat{B}$ to distinguish them from the total effects on the actor (*C*) and the recipient (*B*), may be experienced by neighbors (beyond the actor and recipient) whose coefficient of consanguinity $\hat{Q}$ to the actor differs both from that between the actor and recipient (*Q*) and from that between two random individuals drawn from the larger population ($\bar{Q}$).

From the inelastic population assumption, any increases in fitness must be offset by corresponding decreases in fitness (see also [3]),

1 $$\begin{aligned} -C + B + \hat{B} = 0. \end{aligned}$$

With this, we can calculate the inclusive fitness effect of the behavior, $\Delta W$. Given weak selection, additive genetic effects, and the actor having a coefficient of consanguinity 1 to itself, we simply multiply each term in Eq. (1) by the corresponding measure of consanguinity to obtain

$$\begin{aligned} \Delta W = -1 \cdot C + QB + \hat{Q} \hat{B}. \end{aligned}$$

We can now calculate the constraints under which this behavior will emerge by evaluating $\Delta W > 0$; that is, when the inclusive fitness effect is positive^4,30^. Using the assumption from Eq. (1) that $\hat{B} = C - B$, we have

2 $$\begin{aligned} -C + \frac{Q - \hat{Q}}{1 - \hat{Q}}B > 0. \end{aligned}$$

From inequality (2), we interpret the expression $R = (Q - \hat{Q})/(1 - \hat{Q})$ as a measure of relatedness relative to the competitive neighborhood of the actor^10^. Note that this expression for *R* has two degrees of freedom. Although both are bounded by $0 \le Q, \hat{Q} \le 1$, *R* can take on any value between $-\infty$ and $+1$. As a result, extraordinary spite, where $C > 0$, $B < 0$, and $C > -B$, can evolve. This becomes increasingly likely as the consanguinity between the actor and its neighbors increases, or $\hat{Q} \rightarrow 1$.

A pair of numerical examples, in which the actor pays a cost to harm a recipient who is not kin ($Q = 0$), can illustrate this point. In the first case, the actor is surrounded primarily by close kin ($\hat{Q} = 0.8$). Substituting the values of *Q* and $\hat{Q}$ into inequality (2), we see that the behavior will evolve when $C < -4B$, meaning that selection will favor spite if the actor pays up to four times the cost that it imposes on the recipient. Thus, extraordinary spite can evolve under these conditions (c.f. [﻿^23^]).

In the second case, the actor is surrounded primarily by nonkin ($\hat{Q} = 0.2$). Substituting the values of *Q* and $\hat{Q}$ into inequality (2), we see that the behavior will evolve when $C < -0.25B$, meaning that selection will favor spite only if the actor pays less than one quarte﻿r of the cost that it imposes on the recipient. Thus, only “ordinary” spite can evolve under these conditions.

The sole difference between these two examples is the consanguinity between the actor and its neighbors ($\hat{Q}$), who are the actor’s close kin in the first case but not in the second. And so, because the interaction is spiteful, the respective losses to the actor and recipient become corresponding gains to the actor’s kin in the first case, whereas they become gains to nonkin in the second case. This suggests that appropriately measured background levels of relatedness—that is, measured relative to the actor’s competitive neighborhood—can amplify the effects of spite.

Inequality (2), however, cannot account for extraordinary altruism, because the expression $(Q - \hat{Q})/(1 - \hat{Q})$ can never exceed 1. A further revision to the concept of relatedness is therefore necessary. To this end, we assume that the actor and recipient may compete in different neighborhoods after they interact^10,12,13^, and that the actor’s neighbors may be more genetically similar to the actor than the recipient’s neighbors are to the actor. This results in the partitioning of the fitness effect $\hat{B}$ into its constituent parts: from Eq. (1), a loss of *C* to the actor becomes a gain of *C* to the actor’s neighbors, and a gain of *B* to the recipient becomes a loss of *B* to the recipient’s neighbors. It also results in distinct, neighborhood-specific coefficients of consanguinity: $\hat{Q}_a$, defined as the coefficient between the actor and a different, random individual in the actor’s neighborhood, and $\hat{Q}_r$, defined as the coefficient between the actor and a different, random individual in the recipient’s neighborhood. Again, we can set up an inclusive fitness expression,

$$\begin{aligned} \Delta W = -1 \cdot C + QB + \hat{Q}_a C - \hat{Q}_r B. \end{aligned}$$

Using the condition $\Delta W > 0$, and with some rearrangement, we get

3 $$\begin{aligned} -C + \frac{Q - \hat{Q}_r}{1 - \hat{Q}_a} B > 0. \end{aligned}$$

In contrast to inequality (2), there are now three degrees of freedom in our measure of relatedness^10^, again calculated relative to the appropriate competitive neighborhoods:

4 $$\begin{aligned} R = \frac{Q - \hat{Q}_r}{1 - \hat{Q}_a}. \end{aligned}$$

And, although they are still bounded by $0 \le Q, \hat{Q}_a, \hat{Q}_r \le 1$, *R* can take on *any* real value from $-\infty$ to $+\infty$. Thus, extraordinary altruism, where $C > 0$, $B > 0$, and $C > B$, can evolve alongside extraordinary spite. This becomes increasingly possible as the coefficient of consanguinity between the actor and the actor’s neighbors increases ($\hat{Q}_a \rightarrow 1$) and the coefficient of consanguinity between the actor and the recipient’s neighbors decreases ($\hat{Q}_r \rightarrow 0).$

To illustrate this, consider a pair of cases in which the actor pays a cost to help a recipient who is likely to be close kin ($Q = 0.8$). In the first case, the actor and recipient are surrounded primarily by other close kin ($\hat{Q}_a = 0.8$) but, after their interaction, the recipient disperses to a distant neighborhood and so is surrounded entirely by nonkin ($\hat{Q}_r = 0$). Substituting the values of *Q*, $\hat{Q}_a$, and $\hat{Q}_r$ into inequality (3), the behavior evolves when $C < 4B$, meaning that selection will favor altruism if the actor pays up to four times the benefit that it gives to the recipient. Thus, extraordinary altruism can evolve under these conditions.

In the second case, however, the recipient does not disperse after the interaction, so both the actor and the recipient remain near their shared, close kin ($Q = \hat{Q}_a = \hat{Q}_r = 0.8$). Substituting the values of *Q*, $\hat{Q}_a$, and $\hat{Q}_r$ into inequality (3), the behavior evolves when $C < 0$, meaning that selection will not favor self-sacrificial behavior of any kind. Thus, even “ordinary” altruism cannot evolve under these conditions^9^.

The sole difference between these two examples is the coefficient of consanguinity between the actor and the recipient’s neighbors ($\hat{Q}_r$), who are the actor’s kin in the second case but not in the first. And so, because the interaction is altruistic, the gains to the recipient become corresponding losses to kin in the second case, whereas they become losses to nonkin in the first case. Consequently, background levels of relatedness can amplify the effects of altruism, just as they can for spite.

**Figure 1:**
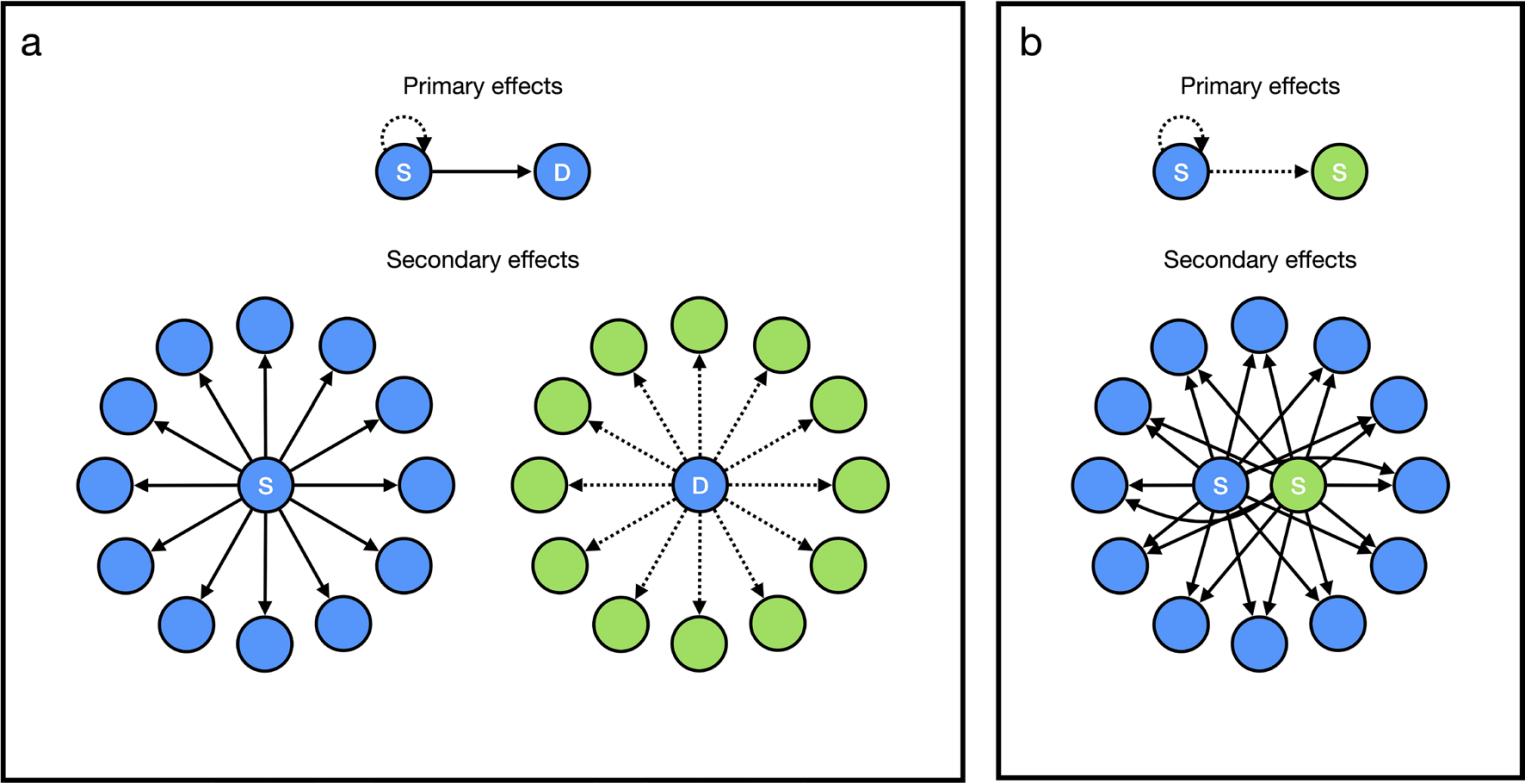
Conditions favoring extraordinary self-sacrifice. Individuals bearing copies of the focal allele are indicated in blue, individuals bearing rival alleles are indicated in green, solid arrows represent benefits, and dashed arrows represent costs. (**a**), On the natal island (top), a sedentary actor (blue “S”) pays a net cost to provide a net benefit to a dispersing recipient bearing a copy of the focal allele (blue “D”). The primary cost to the actor becomes a secondary benefit to the actor’s neighbors on the same island (bottom left), decreasing competition among those bearing copies of the focal allele. Conversely, the primary benefit to the recipient becomes a secondary cost to the recipient’s neighbors on a new island (bottom right), increasing competition among those bearing rival alleles. (**b**), On the natal island (top), a sedentary actor (blue “S”) pays a net cost to impose a net cost on a sedentary recipient bearing a rival allele (green “S”). The primary costs to the actor and recipient become secondary benefits to their neighbors on the same island (bottom), decreasing competition among those bearing copies of the focal allele.

More generally, the secondary effects of social behavior will encourage the evolution of extraordinary self-sacrifice when $|R| > 1$; that is, when the absolute value of the numerator on the right-hand side of Eq. (4) is larger than the denominator. This can occur under circumstances in which the actor is close kin with its neighbors and either: is not close kin with the recipient but is close kin with the recipient’s neighbors; or is close kin with the recipient but is not close kin with the recipient’s neighbors (Fig. 1). As we show below, precisely such circumstances can arise if the actor is able to predict the states of three variables: actor dispersal, recipient dispersal, and kinship. That is, by the coordination of mechanisms designed to gather and use information about the probability of dispersal (“dispersal recognition”) and kinship (“kin recognition”).

## A formal model of extraordinary self-sacrifice

Previous work has found that secondary effects on neighboring kin can suppress the evolution of self-sacrifice (e.g.﻿ [9, 13]). The argument above, however, suggests that secondary effects can sometimes catalyse the evolution of self-sacrifice instead—particularly if the behavior relaxes competition among kin and increases it among nonkin. Indeed, Krupp and Taylor^23^ found that extraordinary spite could evolve under limited dispersal when actors bearing locally common phenotypes interacted with recipients bearing locally rare phenotypes, suggesting that the secondary benefits of spite were passed on to neighboring kin. To formalize this, we leave the generality of Hamilton’s Rule to develop a spatially structured inclusive fitness model that provides circumstances under which extraordinary self-sacrifice will evolve.

Consider a very large, inelastic population of haploid, asexual individuals subdivided into islands of *n* breeders and let *q* be the others-only coefficient of consanguinity between two random individuals born on the same island (SI). A fraction *d* of individuals bears wings, while the remaining fraction does not. All offspring within each island interact in random pairs, giving a net benefit *B* to the recipient at a net cost *C* to self, both of which affect fecundity. Following this, winged individuals disperse to a random island, dying in transit with probability *k*, such that there is a $1-k$ probability of successfully landing on a new island^31^. Next, all individuals produce a large number of offspring and then die. Finally, the offspring compete for one of the breeding vacancies with their neighbors on the same island. Unsuccessful individuals die, and the cycle begins again. We define the fitness of a sedentary individual as $w_{\text {S}} = f_{\text {S}}/f_{\text {tot}}$ and the fitness of a dispersing individual as $w_{\text {D}} = (1-k)f_{\text {D}}/f_{\text {tot}}$, where $f_{\text {S}}$ is the fecundity of a sedentary individual, $f_{\text {D}}$ is the fecundity of a dispersing individual, and $f_{\text {tot}}$ is the fecundity of all individuals on a focal island (SI).

Our model begins under the condition that the actor has no information about dispersal or kinship and, in each subsequent condition, we proceed to give some of this information to the actor: first, whether they have wings; second, whether the recipient has wings; and, third, whether a signal produced prior to interacting identifies the recipient as kin ($Q = 1$) or as nonkin ($Q = 0$). To determine evolutionary stability, we first calculate the inclusive fitness effect of a mutant actor playing $(B+b,C+c)$ for small increments of *b* and *c* and then find the evolutionarily stable marginal cost-benefit ratio, *c*/*b*. We then generate numerical examples of the effects of dispersal and kin recognition on the evolutionarily stable actual cost-benefit ratio, *C*/*B*, defining extraordinary self-sacrifice as $C / |B| > 1$.

**Figure 2:**
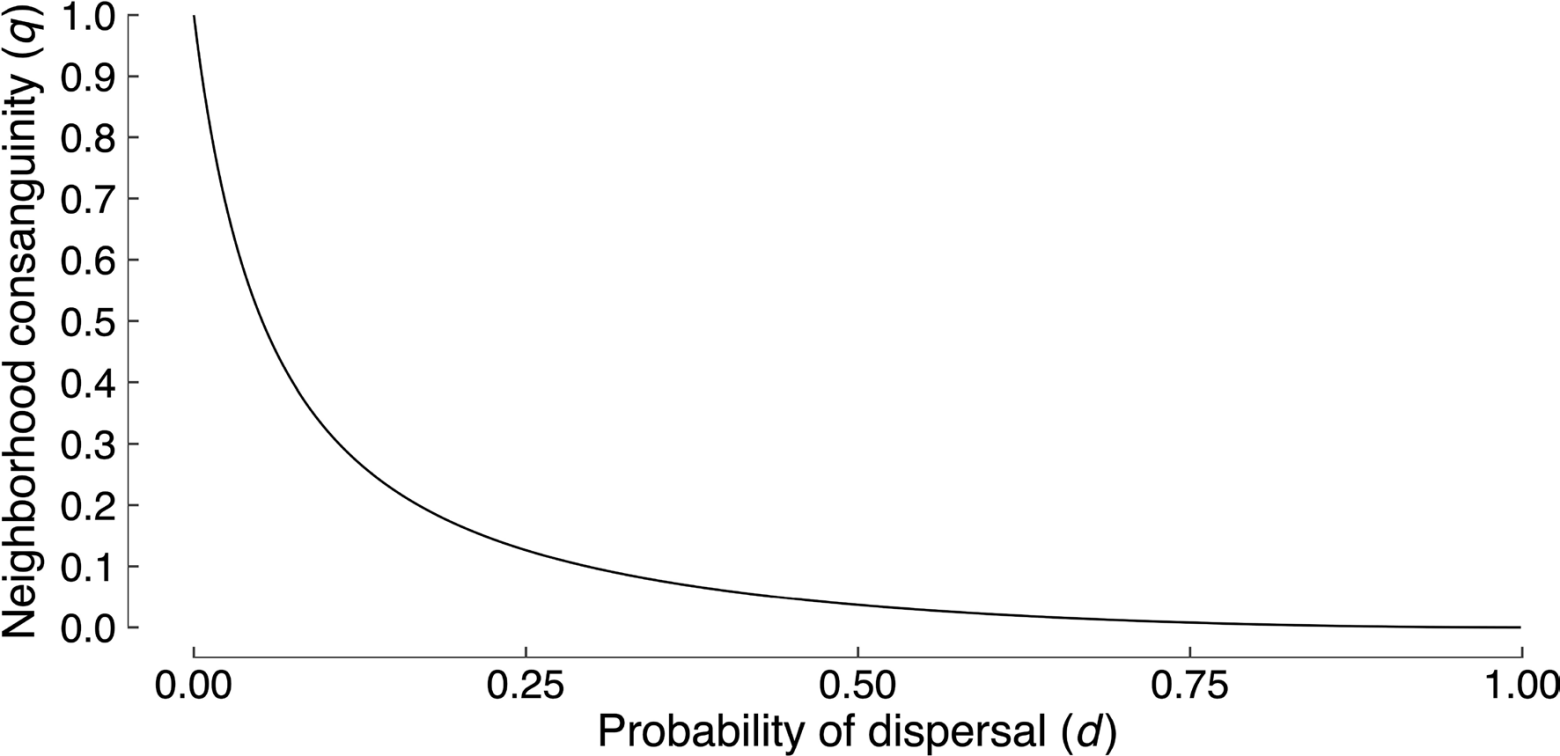
Effect of dispersal rate (*d*) on neighborhood consanguinity (*q*). In the numerical examples, $n = 10$, $k = 0.1$, $q = h^2/(n - h^2(n - 1))$, and $h = (1 - d)/(1 - kd)$.

In these examples, we assume that $n = 10$ and $k = 0.1$, and allow *q* to vary with *d* (eqs. (S1)–(S4)). As shown in Fig. 2, consanguinity among neighbors declines with the dispersal rate. Hence, neighbors are kin under limited dispersal. Further, we assume in the examples that the consequences of each interaction follow a curve of diminishing returns $C = B^2$. We selected such a function because diminishing returns of social behaviour are common in nature^32^. However, they also present a greater challenge to the evolution of extraordinary self-sacrifice in our model than do either linear or accelerating returns, as they disproportionately reduce the impact of actions that entail larger costs to the actor.

**Figure 3:**
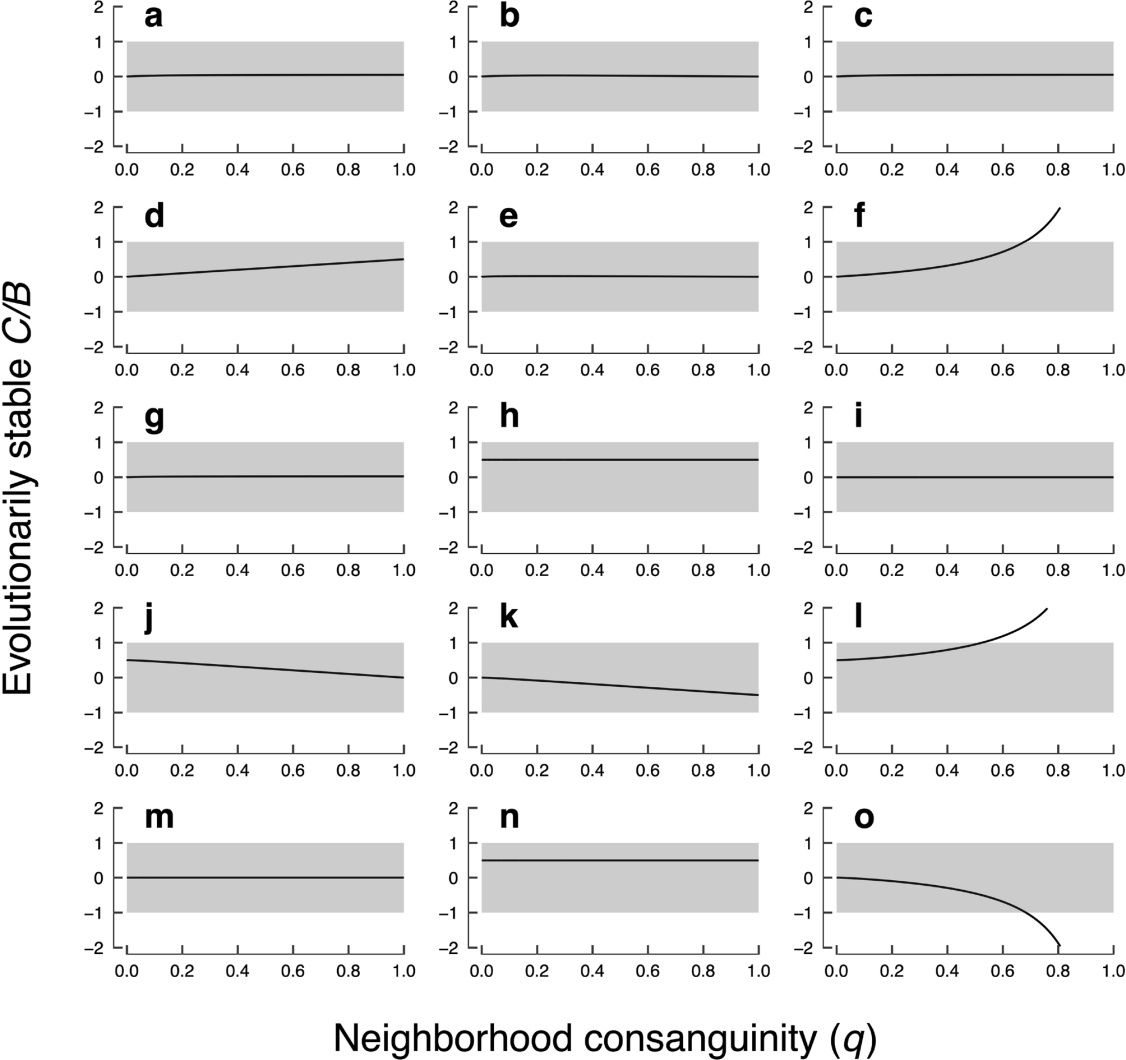
Effect of neighborhood consanguinity (*q*) on evolutionarily stable actual cost-benefit ratios (*C*/*B*), where $n = 10$ and $k = 0.1$. Each panel corresponds to one of the model conditions described in the SI, with results presented at the same scale: (**a**), the actor has no information; (**b**), the actor is dispersing; (**c**), the actor is sedentary; (**d**), both the actor and recipient are dispersing; (**e**), the actor is dispersing but the recipient is sedentary; (**f**), the actor is sedentary but the recipient is dispersing; (**g**), both the actor and recipient are sedentary; (**h**), both the actor and recipient are dispersing, and they produce the same signal; (**i**), both the actor and recipient are dispersing, and they produce different signals; (**j**), the actor is dispersing but the recipient is sedentary, and they produce the same signal; (**k**), the actor is dispersing but the recipient is sedentary, and they produce different signals; (**l**), the actor is sedentary but the recipient is dispersing, and they produce the same signal; (**m**), the actor is sedentary but the recipient is dispersing, and they produce different signals; (**n**), both the actor and recipient are sedentary, and they produce the same signal; (**o**), both the actor and recipient are sedentary, and they produce different signals. Portions of any curve that lie outside the grey windows represent extraordinary self-sacrifice ($C/|B| > 1$).

The complete methods and results are presented in the SI. Here, however, we focus on the three conditions that yield extraordinary self-sacrifice, which can be identified from Figure 3: whereas panels (a)–(e), (g)–(k), (m), and (n) correspond to conditions in which $C / |B| < 1$, panels (f), (l), and (o) correspond to conditions in which $C / |B| > 1$ when neighborhood consanguinity (*q*) is high.

To begin, let us consider the case of a sedentary actor and a dispersing recipient of expected consanguinity $\mathbb {E}(Q) = q$. Let us assume that individuals can reliably infer whether they and their partner will disperse after the interaction by the presence or absence of wings. Sedentary actors will affect the fitness of neighboring kin of consanguinity $\hat{Q}_a = q$ who remain on the natal island with probability $1 - d$. Conversely, dispersing recipients affect competition with neighbors of consanguinity $\hat{Q}_r = \bar{Q} = 0$. This gives the marginal inclusive fitness effect

$$\begin{aligned} \Delta W = -1 \cdot c + qb + (1 - d)qc - 0 \cdot b. \end{aligned}$$

Setting $\Delta W = 0$ and simplifying, the resulting evolutionarily stable marginal cost-benefit ratio is thus

5 $$\begin{aligned} \frac{c}{b} = \frac{q}{1 - (1 - d)q}. \end{aligned}$$

With Eq. (5), we have a case in which extraordinary altruism can evolve: altruism between a sedentary actor and a dispersing recipient is evolutionarily stable for $C/B > 1$ when neighbors are close kin (Fig. 3f).

Two other cases are of particular interest. As above, we assume that actors know whether they and the recipient will disperse after the interaction via the presence or absence of wings. However, we additionally suppose that individuals attempt to match a learned signal of genealogical kinship (SI). In the model, if the actor and recipient produce the same signal, then the coefficient of consanguinity between them is $\mathbb {E}(Q) = 1$, and if they produce different signals, then $\mathbb {E}(Q) = 0$. First, altruism should evolve most easily when the actor is sedentary, the recipient disperses, and the actor and recipient produce the same signal, indicating that they bear identical copies of the focal allele. The marginal inclusive fitness effect of this interaction is

$$\begin{aligned} \Delta W = -1 \cdot c + 1 \cdot b + (1 - d) qc - 0 \cdot b. \end{aligned}$$

Setting $\Delta W = 0$ and simplifying, the evolutionarily stable marginal cost-benefit ratio is

6 $$\begin{aligned} \frac{c}{b} = \frac{1}{1 - (1 - d) q}. \end{aligned}$$

Second, spite should evolve most easily when the actor is sedentary, the recipient is sedentary, and the actor and recipient produce different signals, indicating that they bear rival alleles. The marginal inclusive fitness effect of this interaction is

$$\begin{aligned} \Delta W = -1 \cdot c + 0 \cdot b + (1 - d) qc - (1 - d) qb. \end{aligned}$$

Once more, setting $\Delta W = 0$ and simplifying, the evolutionarily stable marginal cost-benefit ratio is thus

7 $$\begin{aligned} \frac{c}{b} = - \frac{(1 - d) q}{1 - (1 - d) q}. \end{aligned}$$

Panels (l) and (o) of Fig. 3 respectively translate Eqs. (6) and (7) into evolutionarily stable actual cost-benefit ratios, showing that both extraordinary altruism ($C/B > 1$) and extraordinary spite ($C/B < -1$) can evolve. Thus, kin recognition increases the scope for altruism between sedentary actors and dispersing kin and increases the scope for spite between sedentary actors and sedentary nonkin.

## Discussion

We find that individuals can evolve to value others’ fitness more than their own. Specifically, selection favors extraordinary altruism when sedentary actors interact with dispersing kin (Fig. 3f,l), and it favors extraordinary spite when sedentary actors interact with sedentary nonkin (Fig. 3o). Because extraordinary self-sacrifice entails $C > |B|$, the sum of the effects on the actor and recipient is always negative ($B - C < 0$), leading overall to a secondary decrease in competition that can benefit kin. Under limited dispersal, the actor’s neighboring kin benefit secondarily when the actor remains on the natal island. Likewise, the actor’s neighboring kin benefit secondarily from spite when the recipient remains on the actor’s natal island. Finally, in the case of altruism, it is nonkin that pay the price when the recipient arrives on their island. Taken together, we arrive at a simple rule: extraordinary self-sacrifice evolves when an actor’s neighbors are close kin and the recipient’s neighbors are not.

The effects of dispersal and kinship among actors, recipients, and neighbors also become apparent when we consider the conditions of our model that *fail* to favor the evolution of extraordinary self-sacrifice, even when dispersal is limited ($d \rightarrow 0$) and neighborhood consanguinity is high ($q \rightarrow 1$). First, extraordinary self-sacrifice in general does not evolve with a dispersing actor (Fig. 3b,d,e,h–k), because, by dispersing to a new island, the actor gives the secondary benefit of its sacrifice (in the form of reduced competition) to neighbors who are not kin and who therefore bear rival alleles. Second, extraordinary altruism does not evolve with a recipient that is not kin (Fig. 3i,k,m), because this provides a primary benefit to a recipient bearing a rival allele. Third, extraordinary altruism does not evolve with a sedentary recipient (Fig. 3a,c,e,g,j,k,n), because, by remaining on the natal island, the recipient imposes a secondary cost (in the form of increased competition) on neighbors who are the actor’s kin and who therefore bear copies of the focal allele. Fourth, extraordinary spite does not evolve with a recipient that is likely or known to be kin (Fig. 3a–e,g,h,j,n), because this imposes a primary cost on a recipient bearing a copy of the focal allele. Finally, extraordinary spite does not evolve with a dispersing recipient (Fig. 3d,h,i,m), for the same reason that it does not evolve with a dispersing actor: because, by dispersing to a new island, the recipient gives the secondary benefit of the spiteful action (in the form of reduced competition) to neighbors who are not the actor’s kin and who therefore bear rival alleles.

We are aware of two other models that report conditions under which extraordinary self-sacrifice can evolve. The first, by Krupp and Taylor^23^, was briefly discussed above. It also used an inclusive fitness approach set in an island structure, wherein actors could use a signal matching mechanism to distinguish between “native” individuals, whose parents were born on the focal island, and “migrant” individuals, whose parents were born elsewhere. Although actors had no information about dispersal status in their model, dispersal was generally assumed to be rare ($d \rightarrow 0$), causing native actors to be close kin with their neighbors and causing both actors and recipients to be sedentary. Given the close parallels between these conditions and our own (represented in Fig. 3o), it is no surprise that they found that extraordinary spite can evolve among native actors interacting with migrant recipients. Our model extends their analysis, separating the effects of actor and recipient dispersal and making them explicit.

The second model, by McAvoy et al.^33^, used a game theoretic approach set in a heterogeneous social network of *N* individuals, each of whom plays either a “producer” strategy that pays a cost to give a benefit or a “non-producer” strategy that pays no cost and gives no benefit. (Because their approach differs significantly from our own, we have changed their notation and description to better correspond to ours.) One set of games entailed proportional benefits but fixed costs (“pf goods”), wherein a new benefit is given to each connected recipient without additional cost to the actor. Thus, if actor *i* is connnected to $n_i$ recipients, then in games with pf goods, *i* pays $c_i > 0$ only once to give a benefit $b_i > 0$ to each of the $n_i$ recipients. McAvoy et al. found that $c_i > b_i$ can evolve in games with pf goods when there are more connections among individuals in the network than there are individuals themselves. However, this implies that $C < B$, because the net benefit $B = b_i n_i$ grows with the number of connections whereas the net cost $C = c_i$ does not. Consequently, these results do not meet the definition of extraordinary self-sacrifice.

Another set of games in the McAvoy et al. model entailed fixed benefits and fixed costs (“ff goods”), wherein the benefit is divided equally among all connected recipients. Thus, in games with ff goods, the actor pays $c_i$ only once to give a benefit $b_i / n_i$ to each of the $n_i$ recipients. McAvoy et al. found that $c_i > b_i$ can evolve in games with ff goods within “rich-club” networks consisting of a central group of *m* individuals who are connected to each other as well as to a peripheral group of *l* individuals who are connected only to the members of the central group. Under these circumstances, the producer strategy works well for the central group but poorly for the peripheral group; nevertheless, the peripheral group evolves to play the producer strategy. We suspect, however, that this exploitative state of affairs is maintained by a peculiarity of the updating mechanisms of the model, which require individuals to imitate the strategy of better-performing connections, even if it is to their detriment. By playing the producer strategy, the central group *causes* the peripheral group to play the producer strategy as well: central producers have higher payoffs than peripheral non-producers, so peripheral non-producers must update their strategy to produce—despite the fact that it leaves them worse off—because they are connected strictly to better-performing central producers. As the authors show, the central group benefits greatly from this arrangement, particularly as the size of the peripheral group increases, while the peripheral group suffers losses. On the one hand, this implies that $C < B$ for the central group, because the initial cost $c_i$ of the producer strategy to central individuals is more than repaid by the benefits $b_i l / m$ it receives in return for causing peripheral individuals to produce as well; that is, the net cost $C = c_i - b_i l / m$ to a central producer is negative, meaning that it is actually a benefit. On the other hand, this also implies that $C > B$ for the peripheral group, because the net cost to a peripheral producer is $C = c_i$ and the net benefit it gives is $B = b_i$. Thus, production at the periphery would seem to meet the definition of extraordinary self-sacrifice. We wonder, then, if selection would still favor extraordinary self-sacrifice under these conditions if individuals were not powerless to play the strategy that worked best for them, irrespective of the strategy played by their connections.

Hamilton^4^ initially proposed that limited dispersal (in the form of “viscous” populations) would foster the evolution of altruism, because it would give kin the opportunity to interact. Conversely, he suggested that spite would most likely evolve in “dwindling panmictic species”^5^. With the benefit of hindsight, however, we can see that both claims are in need of refinement. From an inclusive fitness perspective, a cost to the actor must be compensated by a benefit to kin, being either the recipient or a neighbor. But limited dispersal also puts these parties in competition with one another, turning altruistic benefits to the former into costs to the latter^9,11,13,34^. As we find here, however, the dilemma of limited dispersal is resolved if the recipient—but not the actor or neighboring kin—can be expected to disperse after the interaction, providing a primary benefit to a consanguineous recipient and imposing a secondary cost on nonkin elsewhere (much as predicted by [9]). Indeed, the high degree of kinship that limited dispersal brings to a neighborhood is essential to the evolution of extraordinary altruism.

Likewise, spite profits not from panmixia but from population structure, because the primary costs to both the actor and the recipient are returned as secondary benefits to the actor’s neighboring kin. However, since limited dispersal increases the chances that individuals interact with kin, actors cannot simply be spiteful to *anyone*^3^. Rather, actors should discriminate along genealogical lines and, although they may not be strictly necessary, kin recognition mechanisms can be helpful in this regard.

Our model makes use of learned kin recognition systems, which are widespread in nature^35–39^. To the extent that kin recognition can operate via other routes, however, our results are not limited to organisms capable of cognition. While there are known theoretical obstacles to the evolution of genetic kin recognition^40,41^, for example, systems such as this have been identified and characterized in several species (e.g.^42–44^). Indeed, allorecognition is common, predates multicellularity, and has independently evolved numerous times^45^.

Notably, our study departs from previous theoretical work on the evolution of self-sacrifice under dispersal and kinship, largely because we ask not only whether individuals might discriminate as a function of kinship but also as a function of both actor and recipient dispersal (see also [22]). Plausibly, sedentary and dispersing types can evolve different degrees of self-interest, such that sedentary individuals generally give more than their dispersing counterparts. This should occur when the actions of sedentary individuals are systematically “funnelled” towards dispersing individuals as a function of organismal physiology or species ecology. In some cases, actors may even cause recipients to develop a dispersing phenotype—for example, by influencing caste determination^46^.

However, our findings also suggest the possibility that, alongside mechanisms of kin recognition, species may have evolved adaptations to estimate the spatial scale of competition^47–49^, such as mechanisms of dispersal recognition. That is, organisms might identify cues of their probability of future competition with social partners or neighboring kin and discriminate accordingly. Certainly, the eusocial insects already provide ample evidence that sedentary and dispersing individuals behave differently. For example, workers of most such species act altruistically (or, in some cases, spitefully) and are sedentary whereas reproductives act selfishly and disperse to found new colonies^19^. Yet, it is unclear whether some mechanism of competition estimation is the cause of such differences. If so, we might expect that individuals can predict the probability of partner dispersal by cues of future dispersal status, such as by chemical signal (e.g.^50^), location within the colony, presence of wings, or body size.

Though this is a knottier problem than we can address here, our results also suggest that aspects of multicellular and colony evolution, such as the division of labor between cell lines and self/nonself discrimination, are a consequence of dispersal patterns and their attendant secondary effects. Sedentary cells may sacrifice themselves to assist dispersing cells in reproducing elsewhere, as can be seen in the social amoeba *Dictyostelium discoideum* which, when starved, aggregates with kin to form a sterile stalk and a reproductive fruiting body^51^. Interestingly, individuals that starve earlier are more likely than those that starve later to become spores^52^. This presents the possibility that signals produced by early starvers to aggregate are attended to because they predict that these same individuals will disperse and compete elsewhere—signals that may be kept honest by virtue of the fact that starvation itself imposes pressure to disperse to find new food sources.

Moreover, sedentary individuals may serve as soldiers or enforcers, ensuring the integrity of the body or colony. For instance, the ascidian *Botryllus schlosseri* operates under limited dispersal, fusing with kin to create a colony with a shared vasculature^53^. However, when individuals encounter nonkin, they produce an immune response that causes damage at the interaction site^42,54^ which, arguably, is a spiteful response to a foreign competitor. Likewise, clones of the polyembryonic parasitoid wasp *Copidosoma floridanum* develop into two distinct castes: soldiers and reproductives. Soldiers grow quickly, spitefully attacking unrelated competitors with specialized mandibles and dying in the host body, whereas reproductives grow more slowly, eventually dispersing to parasitize new hosts^55,56^.

Of course, extraordinary self-sacrifice may evolve more or less easily in the wild than our model suggests. For instance, beyond the assumptions that actors use information about kinship and dispersal, we also assumed diminishing returns of the actor’s behavior, which can make the evolution of extraordinary self-sacrifice more difficult than might some other kinds of cost-benefit relationship. While there are many cases of social systems with diminishing returns^32^, it is possible that some interactions yield linear or even accelerating returns, improving the conditions for extraordinary self-sacrifice. Likewise, whether social goods entail proportional or fixed costs and benefits^33^ may also affect the ease with which extraordinary self-sacrifice evolves.

Even if each of the assumptions made here is met, other factors (such as sexual reproduction) may reduce consanguinity within the neighborhood, working against the evolution of extraordinary self-sacrifice. More generally, extraordinary self-sacrifice may cause significant but rare evolutionary events. This is because the conditions required to support it are themselves likely to be rare, as evidenced by the many scenarios of our model (represented in Fig. 3a–e,g–k,m,n) in which extraordinary self-sacrifice is not evolutionarily stable. Thus, while our model has been productive in demonstrating when and where extraordinary self-sacrifice might arise, further work establishing its prevalence, both theoretical and empirical, is certainly needed. In particular, complementary approaches, such as direct fitness and evolutionary game theoretic methods, may reveal further insights and applications.

## Supplementary Information

Supplementary Information.
