# Supplementary material for: The evolution of extraordinary self-sacrifice
Source: Sci Rep. 2022 Jan 7;12:90. doi: 10.1038/s41598-021-04192-w (PMC8741978; doi:10.1038/s41598-021-04192-w)
Supplement: Supplementary file 1 — Supplementary Information. [file 41598_2021_4192_MOESM1_ESM.pdf]

# Supplementary Information for "The Evolution of Extraordinary Self-sacrifice"

DB Krupp & Wes Maciejewski

# Parent Model

Our parent model, which follows from Taylor [10], is set in an inelastic population large enough that stochastic effects can be ignored. The population is composed of asexual, haploid individuals distributed over a large number of islands, each of which contains $n$ breeding vacancies. A proportion $d$ of all individuals grow wings, and the remaining proportion does not. The stages of the life cycle are:

1. **Interaction.** Individuals are paired at random with a recipient from the same island and pay an expected net fecundity cost $C$ to give an expected net fecundity benefit $B$ to the recipient.
2. **Dispersal.** Winged individuals disperse, dying in transit with probability $k$ or landing on a random island with probability $1-k$.
3. **Breeding.** Individuals produce a large number of offspring, based on the fecundity effects of $B$ and $C$ in stage 1, and then die.
4. **Competition.** Offspring compete for one of the $n$ breeding vacancies on their island. Those that do not secure a vacancy die, and the cycle begins again.

We take the personal fitness of an individual, denoted $w$, to be the expected relative number of their offspring in the next generation. Let us denote the fecundity of dispersing and sedentary individuals as $f_{\mathrm{D}}$ and $f_{\mathrm{S}}$, respectively, and the total fecundity of all individuals on a focal island (which is composed of the weighted sum of all fecundities of island residents, both native and newly-arrived) as $f_{\mathrm{tot}}$. From the above life cycle description, we have the fitness of dispersing and sedentary individuals:

$$w_{\mathrm{D}} = (1-k)\frac{f_{\mathrm{D}}}{f_{\mathrm{tot}}} \text{ and } w_{\mathrm{S}} = \frac{f_{\mathrm{S}}}{f_{\mathrm{tot}}}.$$

To determine the inclusive fitness effects of a social action in this model, we first define two coefficients of consanguinity: that of two different individuals drawn from the same island ($q$) and that of two different individuals drawn from the larger population ($\bar{Q}$). Defining the latter coefficient is straightforward. Because the population is very large, the probability that two randomly selected individuals drawn without replacement from the population bear copies of the focal allele identical by descent is effectively nil, so $\bar{Q} \approx 0$. The former coefficient, however, requires some exposition.

The probability that two randomly selected individuals drawn from the same island, with replacement, bear copies of the focal allele identical by descent is

$$\dot{q} = \frac{1}{n} \cdot 1 + \frac{n-1}{n} \cdot q, \tag{S1}$$

where $q$ is the probability that two randomly selected breeders drawn from the same island, without replacement, bear copies of the focal allele identical by descent. Thus, $\dot{q}$ is a "whole-group" coefficient of consanguinity and $q$ is an "others-only" coefficient of consanguinity [8].

If we let $h$ be the probability that a breeder is native to the island on which it breeds, then $q = h^2 \dot{q}$. With substitution, we can calculate $q$ from the recursion

$$q = h^2 \left( \frac{1}{n} \cdot 1 + \frac{n-1}{n} \cdot q \right). \tag{S2}$$

Expanding and solving for $q$ gives

$$q = \frac{h^2}{n - h^2(n-1)}. \tag{S3}$$

Finally, let us calculate $h$. There is a $1-d$ probability that an individual is sedentary, of a total $1-d$ natives and $(1-k)d$ migrants on the island. Thus,

$$h = \frac{1-d}{1-d+(1-k)d} = \frac{1-d}{1-kd}. \tag{S4}$$

With these definitions, we can now study the effects of actor dispersal, recipient dispersal, and kin recognition on social evolution. In each case, we first calculate the inclusive fitness effect $\Delta W$ of a mutant actor playing $(B+b, C+c)$ for small increments of $b$ and $c$. Next, we find the evolutionarily stable (ES) marginal cost-benefit ratio, $c/b$. Finally, we translate this into an actual cost-benefit ratio, $C/B$. We assume diminishing returns, such that $C = B^2$. Given that $c/b$ can be approximated by the derivative $\mathrm{d}C/\mathrm{d}B$, $c/b = 2C/B$. For instance, if the ES $c/b = 1$, then the ES $C/B = 0.5$. To provide numerical examples of ES actual cost-benefit ratios, we assume that $n = 10$ and $k = 0.1$, and we allow $q$ to vary with $n$, $k$, and $d$, following the arguments above in equations (S1) to (S4). Thus, our model is "closed" with respect to the calculation of consanguinity (Figure 2; see [1]).

We define extraordinary self-sacrifice as $c/|b| > 1$ for the marginal effects and $C/|B| > 1$ for the actual effects. For ease of reference, we summarize the ES marginal cost-benefit ratios for all conditions in Table S1, wherein the highlighted rows refer to conditions under which extraordinary self-sacrifice can evolve. To compare the effects of actor dispersal, recipient dispersal, and kin recognition on ES actual cost-benefit ratios, Figure 3 (main text) presents the numerical examples on the same scale; here, however, we present these examples on case-specific scales that allow us to observe the effects more closely.

To begin, we study the evolution of self-sacrifice in a case in which individuals have no information about actor dispersal, recipient dispersal, or kinship. The results of these initial conditions serve as a benchmark for the results of later conditions. The inclusive fitness effect is composed of the following primary and secondary effects:

- $c$, the net cost to the actor;
- $qb$, the net benefit to the recipient weighted by the coefficient of consanguinity between two different, random individuals drawn from the same island;
- $(1-d)^2qc$, the secondary effect of the cost to the actor weighted by the probability that both the actor and the actor's neighbors remain on the island and by the coefficient of consanguinity between two different, random individuals drawn from the same island;

- $d\bar{Q}c$, the secondary effect of the cost to the actor weighted by the probability that the actor disperses to a new island and by the coefficient of consanguinity between two different, random individuals drawn from the larger population;
- $(1-d)^2qb$, the secondary effect of the benefit to the recipient weighted by the probability that both the recipient and the recipient's neighbors remain on the island and by the coefficient of consanguinity between two different, random individuals drawn from the same island; and
- $d\bar{Q}b$, the secondary effect of the benefit to the recipient weighted by the probability that the recipient disperses to a new island and by the coefficient of consanguinity between two different, random individuals drawn from the larger population.

Putting all of this together, we can write the inclusive fitness effect:

$$\Delta W = -c + qb + (1-d)^2qc + d\bar{Q}c - (1-d)^2qb - d\bar{Q}b.$$

Setting $\Delta W = 0$ and simplifying, the ES marginal cost-benefit ratio is

$$\frac{c}{b} = \frac{q-(1-d)^2q}{1-(1-d)^2q} \tag{S5}$$

and the ES actual cost-benefit ratios are presented numerically in Figures 3a and S1. As both equation (S5) and these figures show, altruism can evolve, but only in the "ordinary" sense.

## Actor dispersal

We now study the effect of actor dispersal. Let us assume that the actor knows whether it will disperse or remain sedentary from the presence or absence of wings, denoting these two phenotypes with D and S, respectively. Thus, the probabilities of actor dispersal ($d$ and $1-d$) can be dropped, as can be any quantities associated with the effects of the alternative actor phenotype. Working from the parent model, then, the inclusive fitness effect of a dispersing actor is

$$\Delta W_{\mathrm{D}} = -c + qb + \bar{Q}c - (1-d)^2qb - d\bar{Q}b,$$

which is ES when $\Delta W_{\mathrm{D}} = 0$. With simplification and rearrangement, this yields

$$\frac{c}{b} = q - (1-d)^2q, \tag{S6}$$

with the ES actual cost-benefit ratios presented numerically in Figures 3b and S2.

Table S1: Effects of actor dispersal, recipient dispersal, and expected consanguinity ($\mathbb{E}(Q)$) on ES marginal cost-benefit ratios

| Actor dispersal | Recipient dispersal | $\mathbb{E}(Q)$ | Evolutionarily stable $c/b$ |
|---|---|---|---|
| $d$ | $d$ | $q$ | $\frac{q-(1-d)^2q}{1-(1-d)^2q}$ |
| Dispersing | $d$ | $q$ | $q-(1-d)^2q$ |
| Sedentary | $d$ | $q$ | $\frac{q-(1-d)^2q}{1-(1-d)q}$ |
| Dispersing | Dispersing | $q$ | $q$ |
| Dispersing | Sedentary | $q$ | $q-(1-d)q$ |
| Sedentary | Dispersing | $q$ | $\frac{q}{1-(1-d)q}$ |
| Sedentary | Sedentary | $q$ | $\frac{q-(1-d)q}{1-(1-d)q}$ |
| Dispersing | Dispersing | 1 | 1 |
| Dispersing | Dispersing | 0 | 0 |
| Dispersing | Sedentary | 1 | $1-(1-d)q$ |
| Dispersing | Sedentary | 0 | $-(1-d)q$ |
| Sedentary | Dispersing | 1 | $\frac{1}{1-(1-d)q}$ |
| Sedentary | Dispersing | 0 | 0 |
| Sedentary | Sedentary | 1 | 1 |
| Sedentary | Sedentary | 0 | $-\frac{(1-d)q}{1-(1-d)q}$ |

Notes: $\mathbb{E}(Q)$ refers to the expected coefficient of consanguinity between the actor and the recipient, given the information available (i.e., with versus without kin recognition). Highlighted rows correspond to conditions under which extraordinary self-sacrifice can evolve.

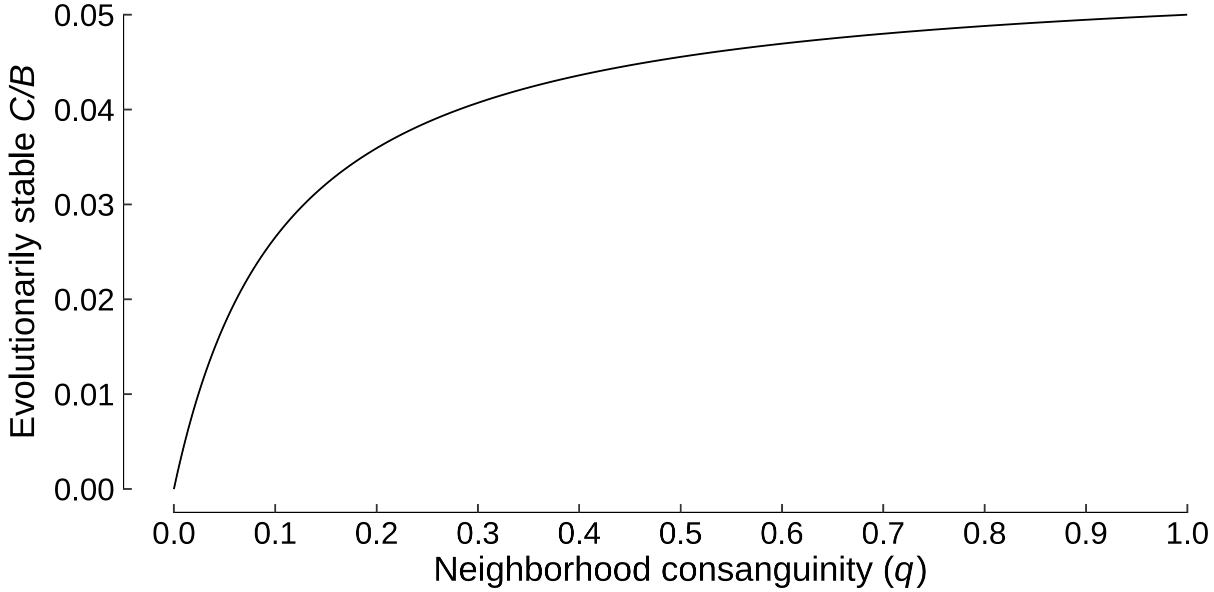


Figure S1: Effect of neighborhood consanguinity ($q$) on ES actual cost-benefit ratios ($C/B$) when the actor has no information about dispersal or kinship.

Conversely, the inclusive fitness effect of a sedentary actor is

$$\Delta W_{\mathrm{S}} = -c + qb + (1-d)qc - (1-d)^2 qb - d\bar{Q}b,$$

which is ES when $\Delta W_{\mathrm{S}} = 0$. With simplification and rearrangement, this yields

$$\frac{c}{b} = \frac{q-(1-d)^2 q}{1-(1-d)q}, \tag{S7}$$

with the ES actual cost-benefit ratios presented numerically in Figures 3c and S3.

Let us now compare equations (S6) and (S7), which differ only in their denominators. When neighborhood consanguinity is high (i.e., $d \to 0$, $q \to 1$), the ES $c/b$ for a sedentary actor will be larger than that of a dispersing actor. Thus, altruism will tend to evolve more easily when actors are sedentary (Figures 3c and S3) than when they disperse (Figures 3b and S2). This is because a sedentary actor's sacrifice relaxes competition among genealogical kin, whereas a dispersing actor's sacrifice has no such effect. Consequently, sedentary actors have a greater incentive than dispersing actors to be selfless—especially if some of the actor's recipients are likely to disperse, imposing the secondary costs of the actor's altruism on nonkin elsewhere. Still, extraordinary self-sacrifice does not evolve under such conditions alone.

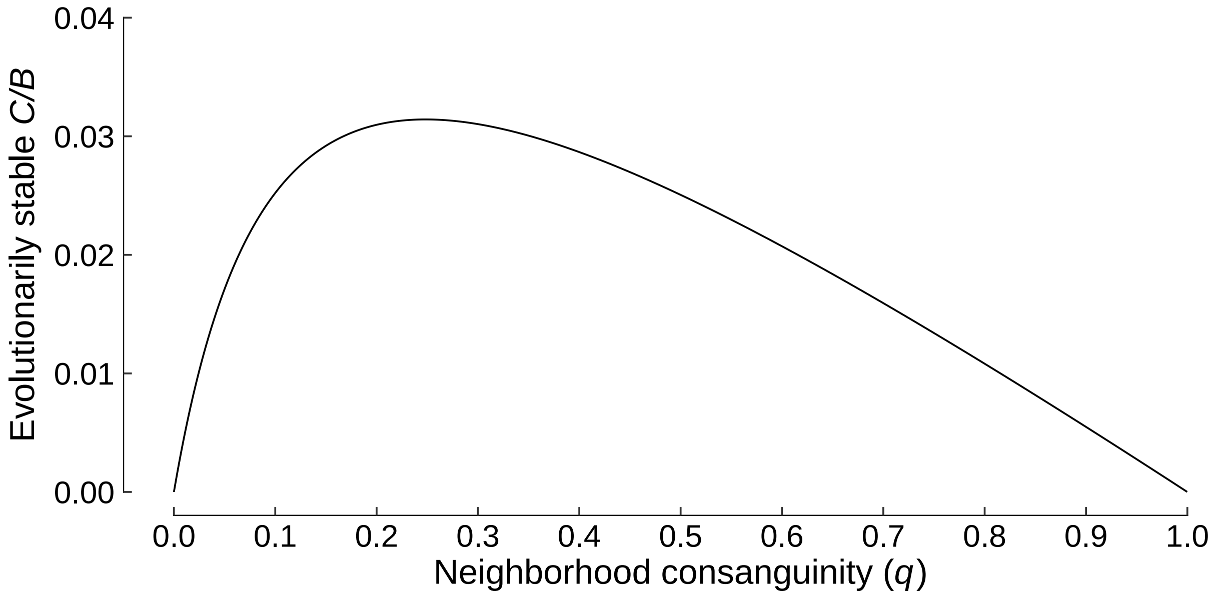


Figure S2: Effect of neighborhood consanguinity ($q$) on ES actual cost-benefit ratios ($C/B$) for a dispersing actor.

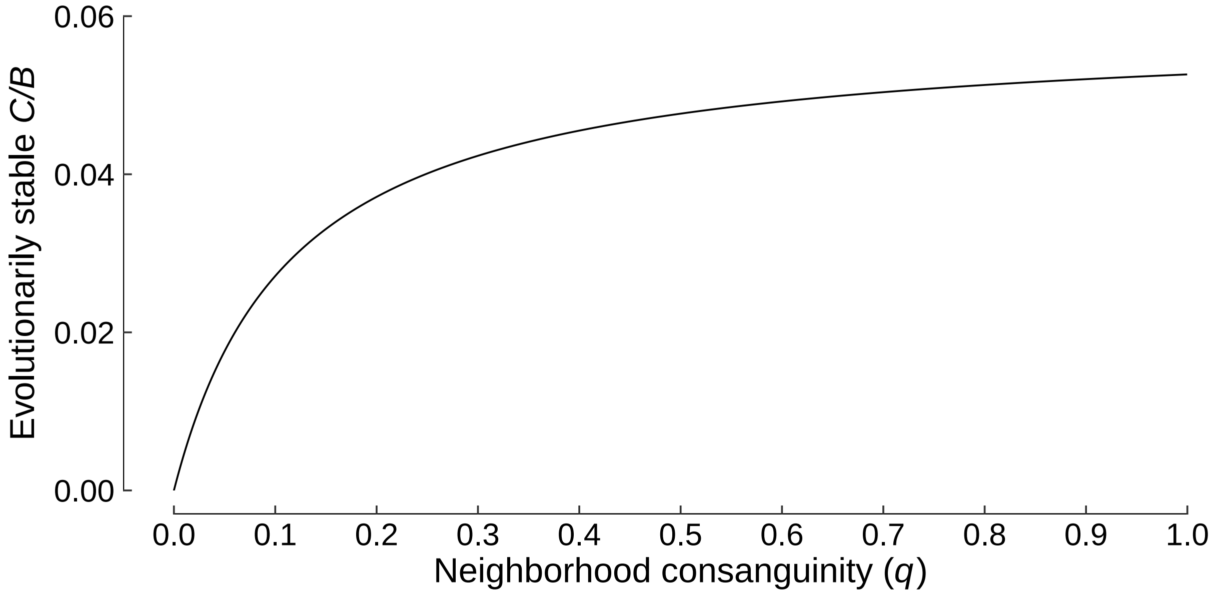


Figure S3: Effect of neighborhood consanguinity ($q$) on ES actual cost-benefit ratios ($C/B$) for a sedentary actor.

## Recipient dispersal

Next, we study the effect of recipient dispersal. As above, we assume that the actor knows whether it will disperse. However, by recognizing whether the recipient bears wings, it

can determine whether the recipient will also disperse. Thus, the probabilities of recipient dispersal ($d$ and $1-d$) can also be dropped, as can be any quantities associated with the effects of the alternative recipient phenotype. First, let us consider a dispersing actor interacting with a dispersing recipient, denoted DD. Here, the inclusive fitness effect is

$$\Delta W_{\mathrm{DD}} = -c + qb + \bar{Q}c - \bar{Q}b,$$

which is ES when $\Delta W_{\mathrm{DD}} = 0$. With simplification and rearrangement, this gives the ES marginal cost-benefit ratio

$$\frac{c}{b} = q, \tag{S8}$$

with the ES actual cost-benefit ratios presented numerically in Figures 3d and S4. This is Hamilton's (1964) classic result for the evolution of altruism [2]. Note, however, that because $0 \leq q \leq 1$, the cost of altruism to a dispersing actor cannot exceed the benefit to a dispersing recipient.

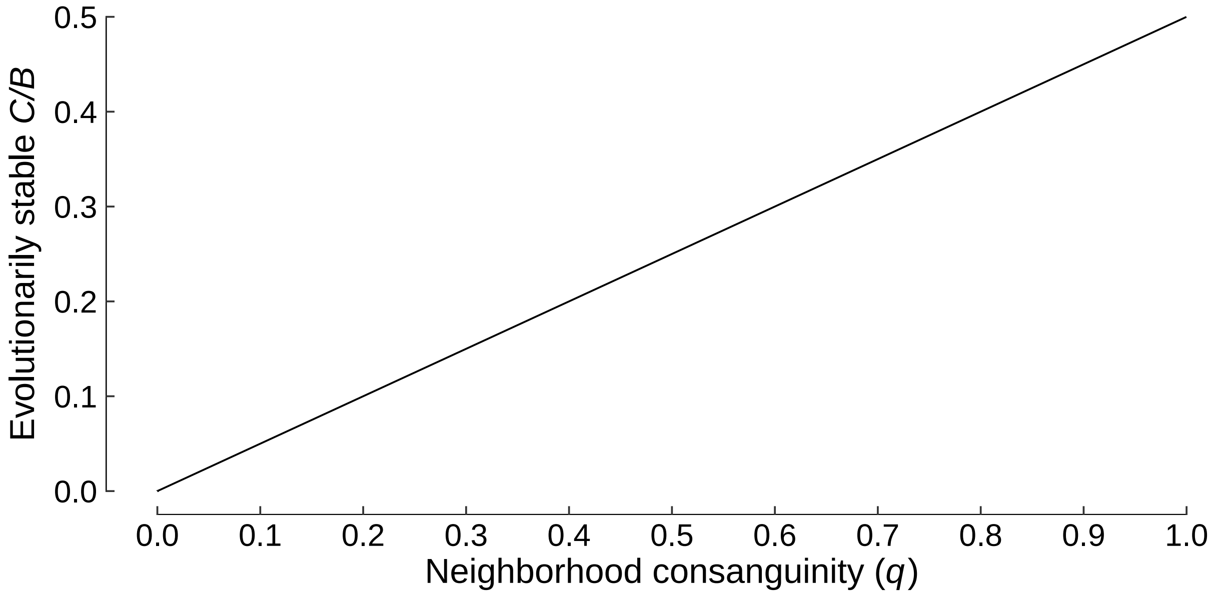


Figure S4: Effect of neighborhood consanguinity ($q$) on ES actual cost-benefit ratios ($C/B$) for a dispersing actor and a dispersing recipient.

Second, let us consider a dispersing actor interacting with a sedentary recipient, denoted DS. The inclusive fitness effect is

$$\Delta W_{\mathrm{DS}} = -c + qb + \bar{Q}c - (1-d)qb,$$

which is ES when $\Delta W_{\mathrm{DS}} = 0$. This gives the ES marginal cost-benefit ratio

$$\frac{c}{b} = q - (1-d)q, \tag{S9}$$

with the ES actual cost-benefit ratios presented numerically in Figures 3e and S5.

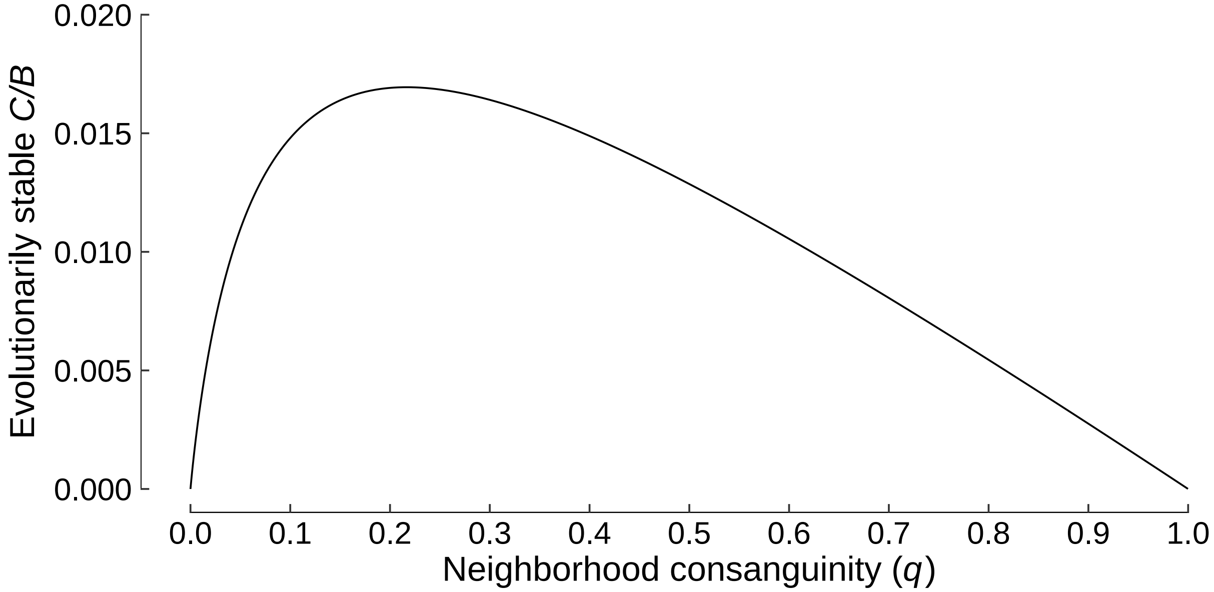


Figure S5: Effect of neighborhood consanguinity ($q$) on ES actual cost-benefit ratios ($C/B$) for a dispersing actor and a sedentary recipient.

Third, let us consider a sedentary actor interacting with a dispersing recipient, denoted SD. The inclusive fitness effect is

$$\Delta W_{\mathrm{SD}} = -c + qb + (1-d)qc - \bar{Q}b,$$

which is ES when $\Delta W_{\mathrm{SD}} = 0$. This gives the ES marginal cost-benefit ratio

$$\frac{c}{b} = \frac{q}{1-(1-d)q}, \tag{S10}$$

with the ES actual cost-benefit ratios presented numerically in Figures 3f and S6.

Finally, let us consider a sedentary actor interacting with a sedentary recipient, denoted SS. The inclusive fitness effect is

$$\Delta W_{\mathrm{SS}} = -c + qb + (1-d)qc - (1-d)qb,$$

which is ES when $\Delta W_{\mathrm{SS}} = 0$. This gives the ES marginal cost-benefit ratio

$$\frac{c}{b} = \frac{q-(1-d)q}{1-(1-d)q}, \tag{S11}$$

with the ES actual cost-benefit ratios presented numerically in Figures 3g and S7.

As can be seen, the largest ES $c/b$ is for the condition of a sedentary actor interacting with a dispersing recipient (equation (S10)). This condition can exceed both the marginal $c/b \leq 1$ and actual $C/B \leq 1$ limits (Figures 3f and S6). Conversely, the smallest cost-benefit ratios arise under the condition of a dispersing actor and a sedentary recipient (equation (S9) and Figures 3e and S5). Thus, altruism evolves most easily when actors are sedentary and recipients disperse.

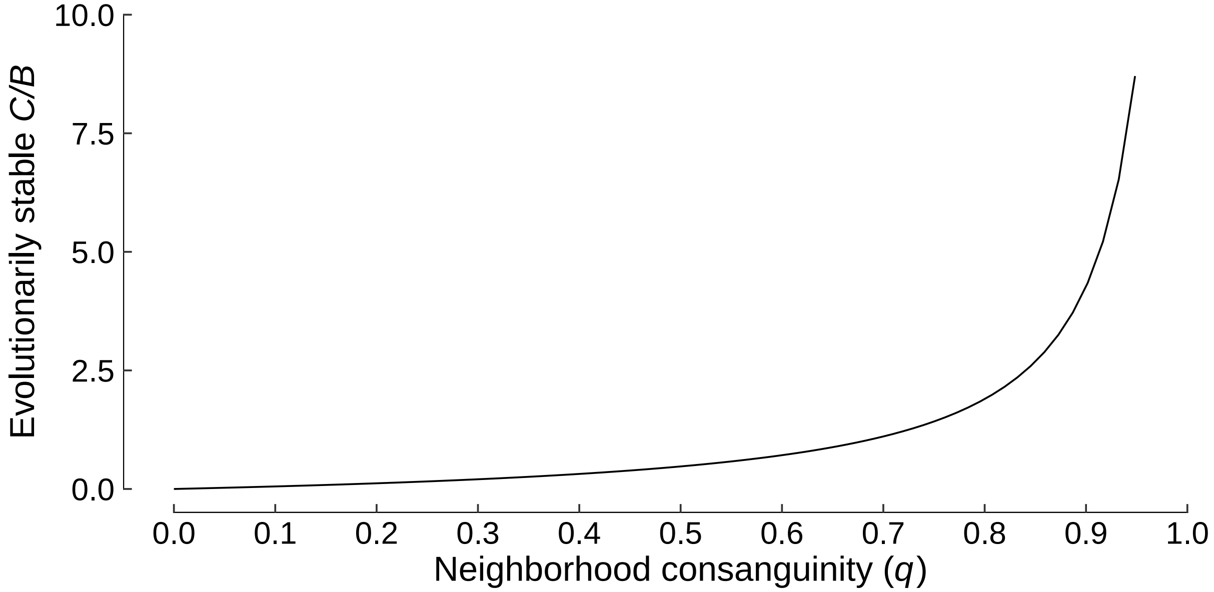


Figure S6: Effect of neighborhood consanguinity ($q$) on ES actual cost-benefit ratios ($C/B$) for a sedentary actor and a dispersing recipient. The $y$-axis is truncated at $C/B = 10$ but, in this numerical example, the maximum ES $C/B \approx 2632$ as $q \to 1$.

## Kin Recognition

Finally, we study the effect of kin recognition. As above, the actor knows its own dispersal type and that of the recipient. However, we additionally suppose that individuals bear a kin recognition mechanism known as *phenotype matching* [3–6, 9, 11]. Following Krupp and Taylor [7], individuals produce a signal that is correlated with consanguinity. The stages of the signaling process are as follows:

1. **Learning.** After birth, offspring learn a signal produced by their parent;
2. **Production.** After being paired with their partner, but prior to their interaction, each individual produces the signal they learned; and
3. **Generation.** After the interaction stage but before dispersal, all individuals discard the signal learned in stage 1 and generate a new, shared signal unique to their island.

As a result, parents pass on the signal of their natal island to their offspring (stage 1), and offspring produce this signal prior to interaction (stage 2). Assuming that dispersal is rare, an island may thus have two signals: a "native" signal produced by most individuals and a "migrant" signal produced by a minority. An immigrant who becomes a breeder has offspring that produce the migrant signal, but any of those offspring who later become breeders will teach the native signal to their own offspring (stage 3).

With this, individuals can estimate the consanguinity between themselves and their partners, $\mathbb{E}(Q)$. For simplicity, let us assume that island-specific signals have vanishingly small variance, so that native and migrant signals do not overlap. Consequently, if two individu-

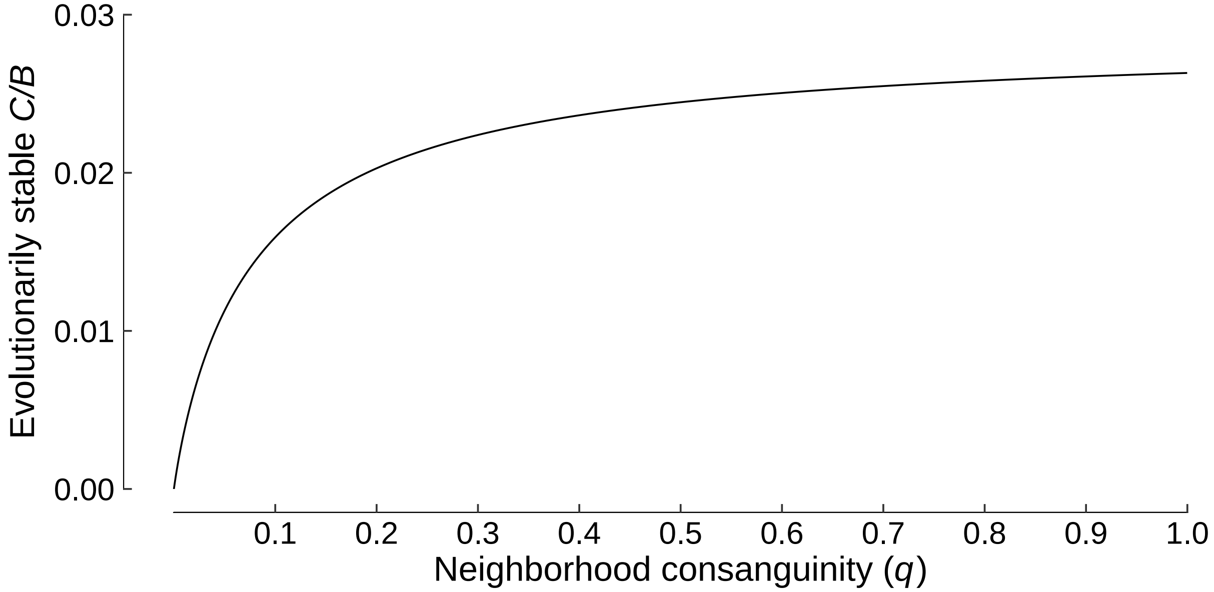


Figure S7: Effect of neighborhood consanguinity ($q$) on ES actual cost-benefit ratios ($C/B$) for a sedentary actor and a sedentary recipient.

als produce the same signal, then the coefficient of consanguinity $\mathbb{E}(Q)$ between them is 1. Conversely, if they produce different signals, then $\mathbb{E}(Q) = 0$.

First, let us consider a dispersing actor interacting with a dispersing recipient who produces the same signal, denoted DD|1. The inclusive fitness effect is

$$\Delta W_{\mathrm{DD}|1} = -c + 1 \cdot b + \bar{Q}c - \bar{Q}b,$$

which is ES when $\Delta W_{\mathrm{DD}|1} = 0$. With simplification and rearrangement, this gives the ES marginal cost-benefit ratio

$$\frac{c}{b} = 1, \tag{S12}$$

with the ES actual cost-benefit ratios presented numerically in Figures 3h and S8.

Second, let us consider a dispersing actor interacting with a dispersing recipient who produces a different signal, denoted DD|0. The inclusive fitness effect is

$$\Delta W_{\mathrm{DD}|0} = -c + 0 \cdot b + \bar{Q}c - \bar{Q}b,$$

which is ES when $\Delta W_{\mathrm{DD}|0} = 0$. This gives the ES marginal cost-benefit ratio

$$\frac{c}{b} = 0, \tag{S13}$$

with the ES actual cost-benefit ratios presented numerically in Figures 3i and S9.

Third, let us consider a dispersing actor interacting with a sedentary recipient who produces the same signal, denoted DS|1. The inclusive fitness effect is

$$\Delta W_{\mathrm{DS}|1} = -c + 1 \cdot b + \bar{Q}c - (1-d)qb,$$

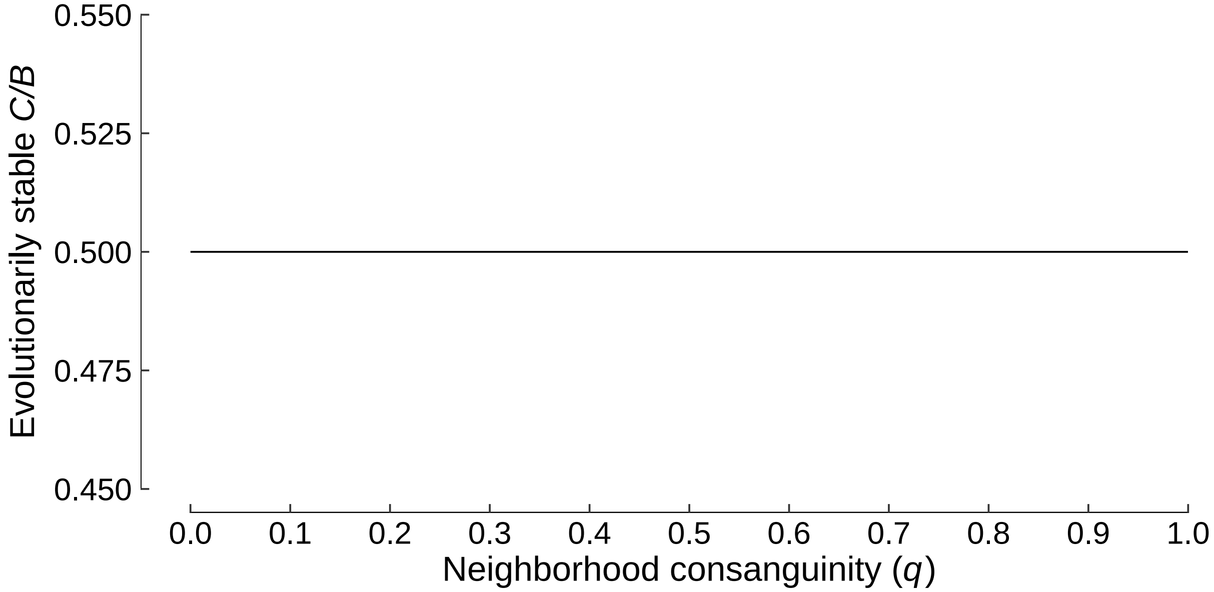


Figure S8: Effect of neighborhood consanguinity ($q$) on ES actual cost-benefit ratios ($C/B$) for a dispersing actor and a dispersing recipient that produce the same signal.

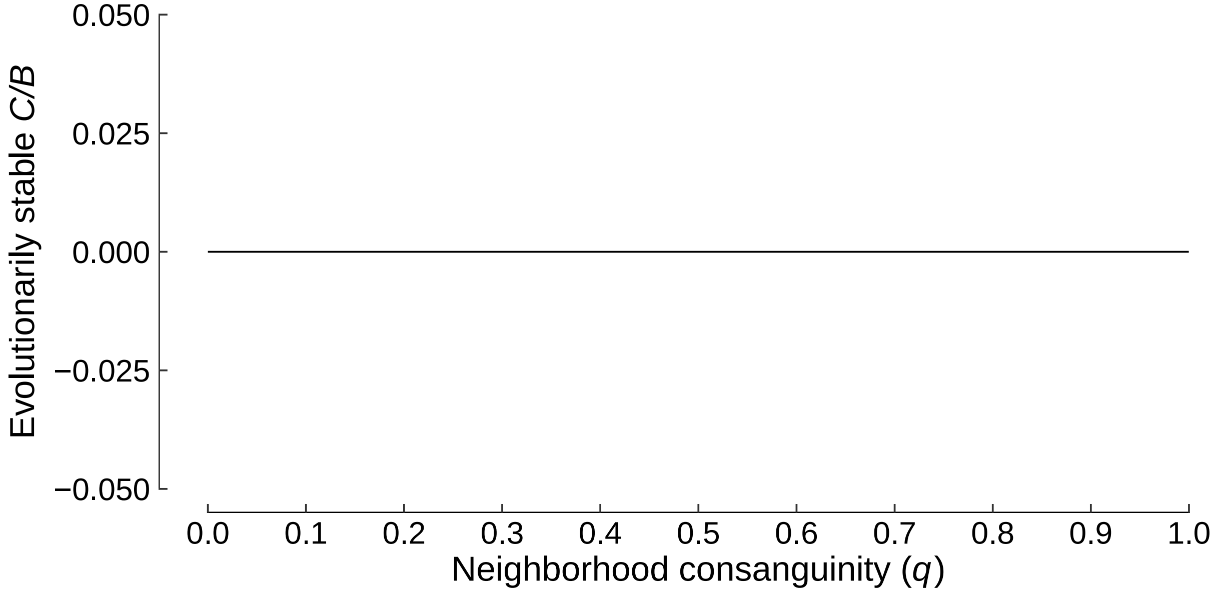


Figure S9: Effect of neighborhood consanguinity ($q$) on ES actual cost-benefit ratios ($C/B$) for a dispersing actor and a dispersing recipient that produce different signals.

which is ES when $\Delta W_{\mathrm{DS}|1} = 0$. This gives the ES marginal cost-benefit ratio

$$\frac{c}{b} = 1 - (1 - d)q, \tag{S14}$$

with the ES actual cost-benefit ratios presented numerically in Figures 3j and S10.

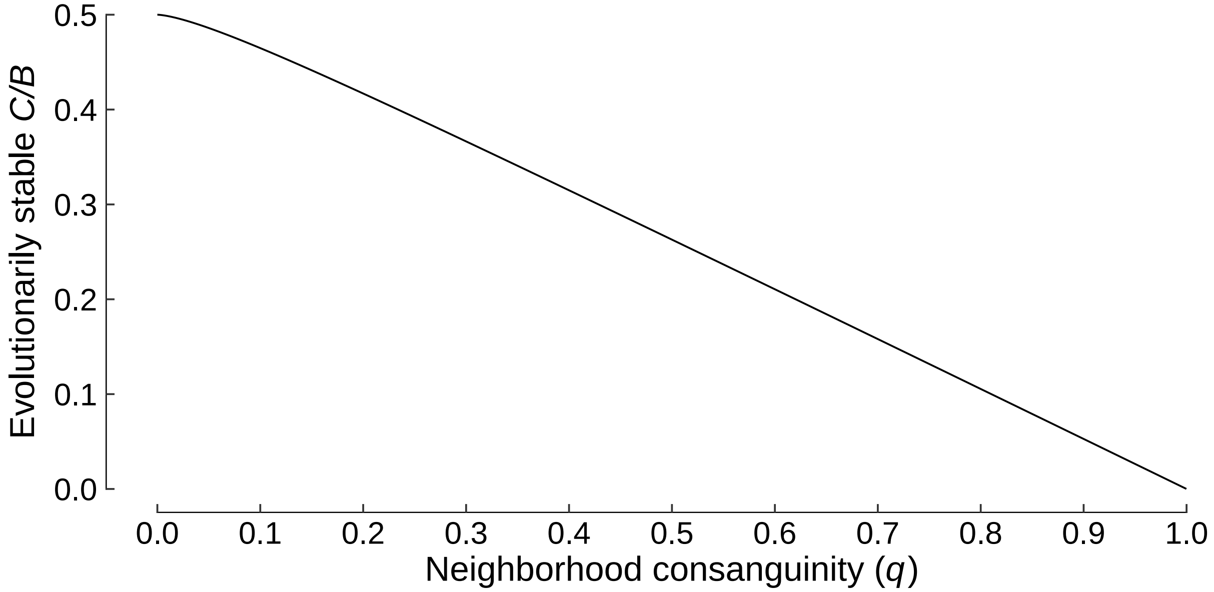


Figure S10: Effect of neighborhood consanguinity ($q$) on ES actual cost-benefit ratios ($C/B$) for a dispersing actor and a sedentary recipient that produce the same signal.

Fourth, let us consider a dispersing actor interacting with a sedentary recipient who produces a different signal, denoted DS|0. The inclusive fitness effect is

$$\Delta W_{\mathrm{DS}|0} = -c + 0 \cdot b + \bar{Q}c - (1-d)qb,$$

which is ES when $\Delta W_{\mathrm{DS}|0} = 0$. This gives the ES marginal cost-benefit ratio

$$\frac{c}{b} = -(1-d)q, \tag{S15}$$

with the ES actual cost-benefit ratios presented numerically in Figures 3k and S11.

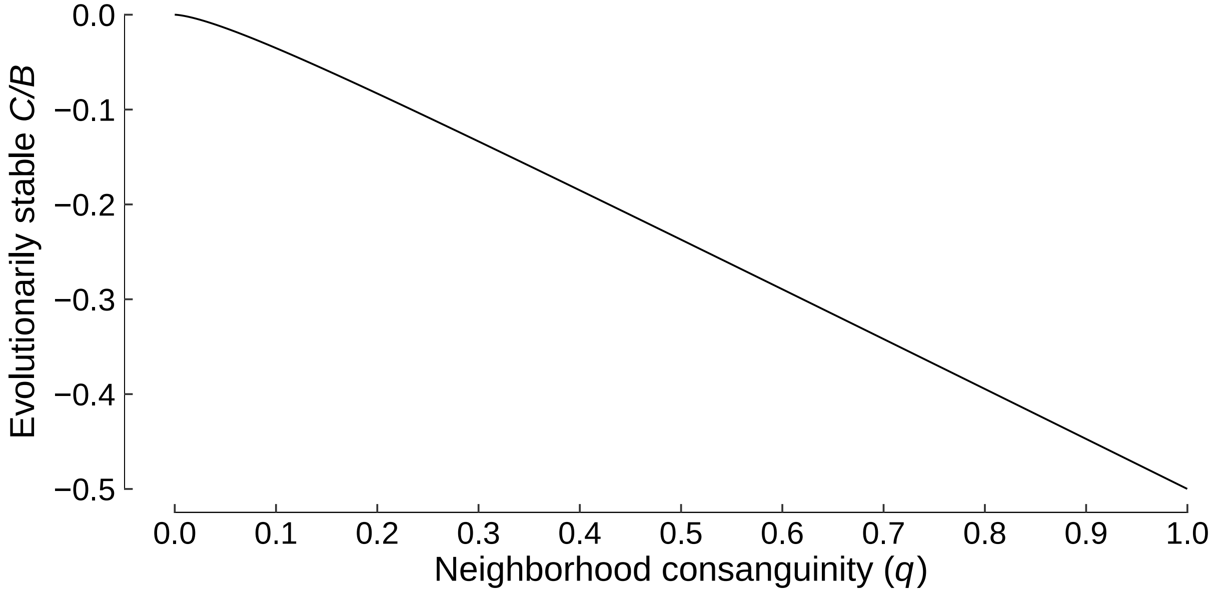


Figure S11: Effect of neighborhood consanguinity ($q$) on ES actual cost-benefit ratios ($C/B$) for a dispersing actor and a sedentary recipient that produce different signals.

Fifth, let us consider a sedentary actor interacting with a dispersing recipient who produces the same signal, denoted SD|1. The inclusive fitness effect is

$$\Delta W_{\mathrm{SD}|1} = -c + 1 \cdot b + (1-d)qc - \bar{Q}b,$$

which is ES when $\Delta W_{\mathrm{SD}|1} = 0$. This gives the ES marginal cost-benefit ratio

$$\frac{c}{b} = \frac{1}{1-(1-d)q}, \tag{S16}$$

with the ES actual cost-benefit ratios presented numerically in Figures 3l and S12.

Sixth, let us consider a sedentary actor interacting with a dispersing recipient who produces a different signal, denoted SD|0. The inclusive fitness effect is

$$\Delta W_{\mathrm{SD}|0} = -c + 0 \cdot b + (1-d)qc - \bar{Q}b,$$

which is ES when $\Delta W_{\mathrm{SD}|0} = 0$. This gives the ES marginal cost-benefit ratio

$$\frac{c}{b} = 0, \tag{S17}$$

with the ES actual cost-benefit ratios presented numerically in Figures 3m and S13.

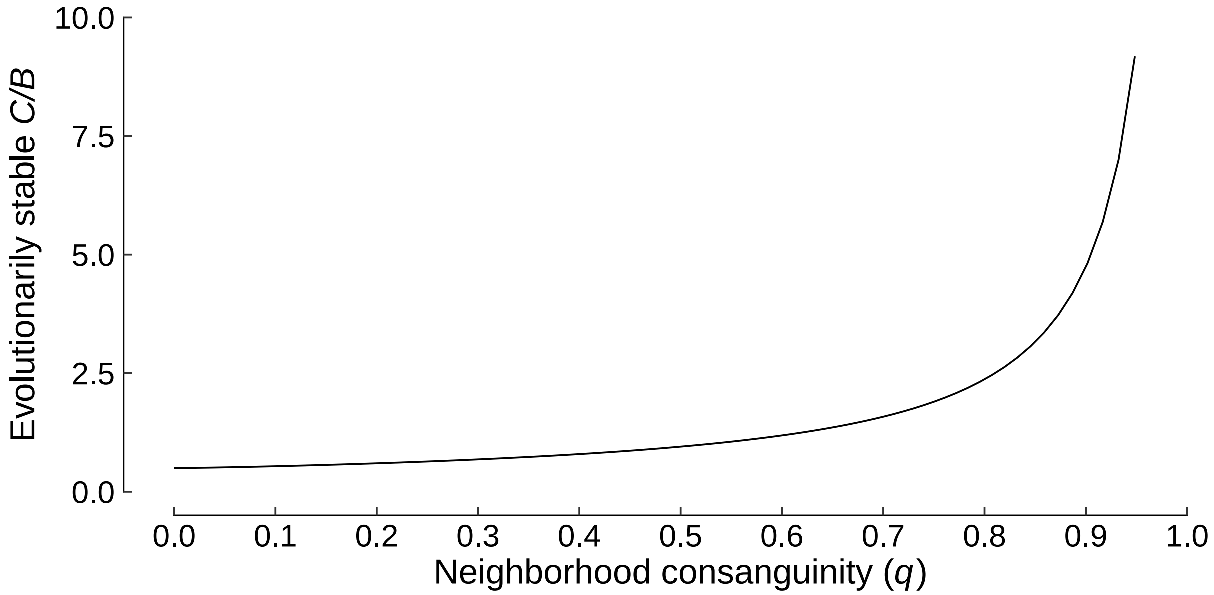


Figure S12: Effect of neighborhood consanguinity ($q$) on ES actual cost-benefit ratios ($C/B$) for a sedentary actor and a dispersing recipient that produce the same signal. The $y$-axis is truncated at $C/B = 10$ but, in this numerical example, the maximum ES $C/B \approx 2632$ as $q \to 1$.

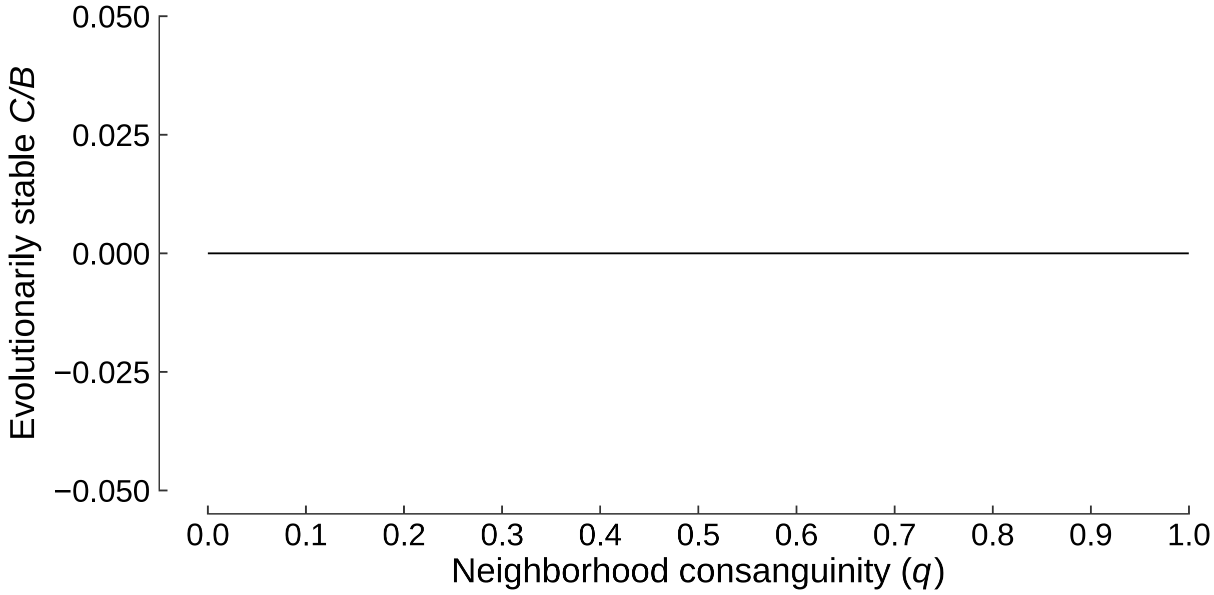


Figure S13: Effect of neighborhood consanguinity ($q$) on ES actual cost-benefit ratios ($C/B$) for a sedentary actor and a dispersing recipient that produce different signals.

Seventh, let us consider a sedentary actor interacting with a sedentary recipient who produces

the same signal, denoted SS|1. The inclusive fitness effect is

$$\Delta W_{\mathrm{SS}|1} = -c + 1 \cdot b + (1-d)qc - (1-d)qb,$$

which is ES when $\Delta W_{\mathrm{SS}|1} = 0$. This gives the ES marginal cost-benefit ratio

$$\frac{c}{b} = 1, \tag{S18}$$

with the ES actual cost-benefit ratios presented numerically in Figures 3n and S14.

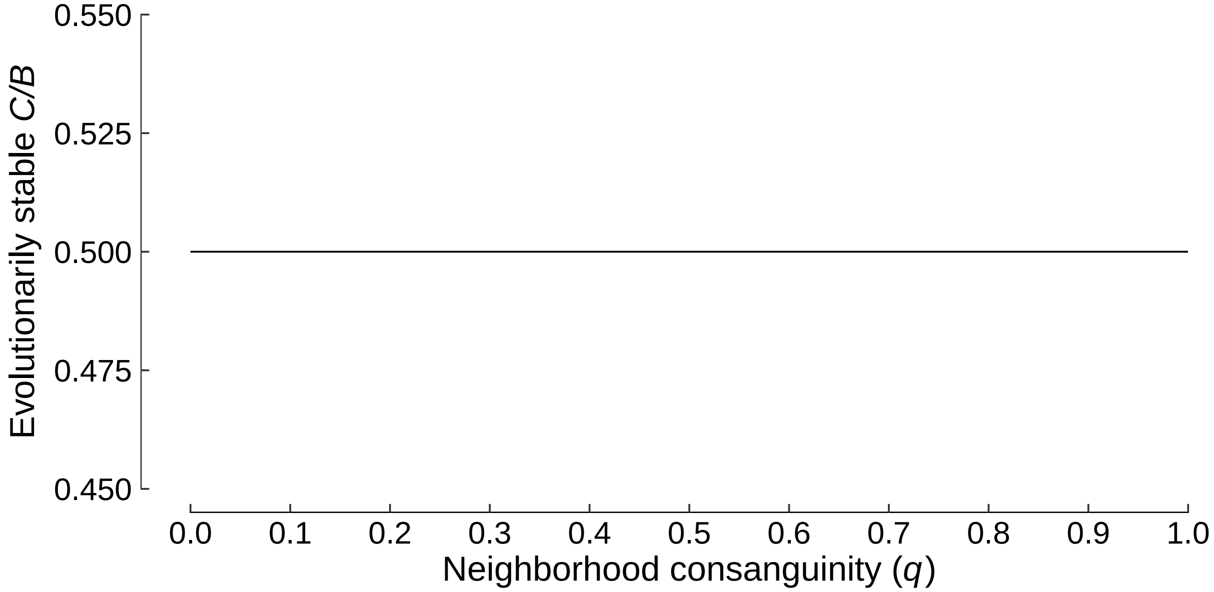


Figure S14: Effect of neighborhood consanguinity ($q$) on ES actual cost-benefit ratios ($C/B$) for a sedentary actor and a sedentary recipient that produce the same signal.

Finally, let us consider a sedentary actor interacting with a sedentary recipient who produces a different signal, denoted SS|0. The inclusive fitness effect is

$$\Delta W_{\mathrm{SS}|0} = -c + 0 \cdot b + (1-d)qc - (1-d)qb,$$

which is ES when $\Delta W_{\mathrm{SS}|0} = 0$. This gives the ES marginal cost-benefit ratio

$$\frac{c}{b} = -\frac{(1-d)q}{1-(1-d)q}, \tag{S19}$$

with the ES actual cost-benefit ratios presented numerically in Figures 3o and S15.

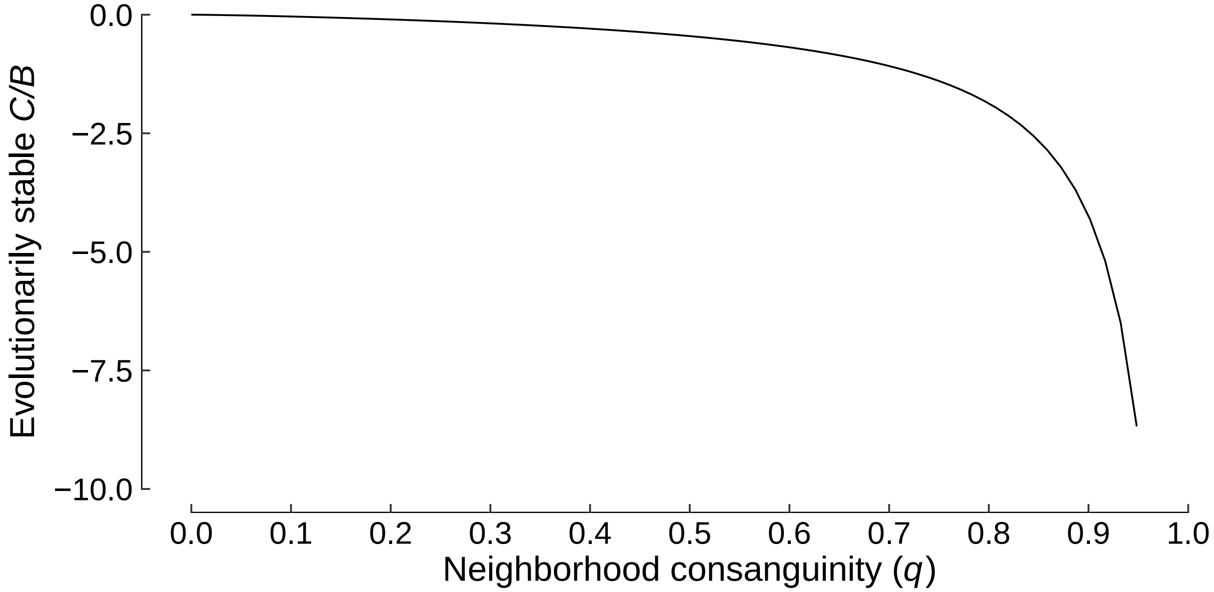


Figure S15: Effect of neighborhood consanguinity ($q$) on ES actual cost-benefit ratios ($C/B$) for a sedentary actor and a sedentary recipient that produce different signals. The $y$-axis is truncated at $C/B = -10$ but, in this numerical example, the minimum ES $C/B \approx -2632$ as $q \to 1$.

With the introduction of kin recognition, we now have two conditions that can exceed both the $c/|b| \leq 1$ and $C/|B| \leq 1$ limits. The first, in which a sedentary actor interacts with dispersing kin (equation (S16)), allows for the evolution of extraordinary altruism—a result nearly identical to that of equation (S10), which was obtained without the use of kin recognition. The second, in which a sedentary actor interacts with sedentary nonkin (equation (S19)), allows for the evolution of extraordinary spite. The latter condition offers a useful contrast to interactions with dispersing nonkin (equations (S13) and (S17)): neither altruism nor spite evolves under such conditions, because the primary cost borne by the recipient will not be translated into a secondary benefit to the actor's kin.

# References

[1] Cooper, G. A., S. R. Levin, G. Wild, and S. A. West (2018). Modeling relatedness and demography in social evolution. *Evolution Letters 2*(4), 260–271.

[2] Hamilton, W. (1964). The genetical evolution of social behaviour (I and II). *Journal of Theoretical Biology 7*, 1–52.

[3] Hauber, M. E. and P. W. Sherman (2001, October). Self-referent phenotype matching: Theoretical considerations and empirical evidence. *Trends in Neurosciences 24*(10), 609–616.

[4] Hepper, P. G. (1991). *Kin Recognition*. New York: Cambridge University Press.

[5] Krupp, D., L. M. DeBruine, and B. C. Jones (2011). Cooperation and conflict in the light of kin recognition systems. In C. A. Salmon and T. K. Shackelford (Eds.), *The Oxford Handbook of Evolutionary Family Psychology*, pp. 345–364. New York: Oxford University Press.

[6] Krupp, D. B. and P. D. Taylor (2013). Enhanced kin recognition through population estimation. *American Naturalist 181*(5), 707–714.

[7] Krupp, D. B. and P. D. Taylor (2015, May). Social evolution in the shadow of asymmetrical relatedness. *Proceedings of the Royal Society of London B: Biological Sciences 282*(1807), 20150142.

[8] Pepper, J. W. (2000). Relatedness in trait group models of social evolution. *Journal of Theoretical Biology 206*(3), 355–368.

[9] Sherman, P., H. Reeve, and D. W. Pfennig (1997). Recognition systems. In J. Krebs and N. B. Davies (Eds.), *Behavioural Ecology* (Fourth ed.)., pp. 69–96. Oxford: Backwell.

[10] Taylor, P. D. (1992). Altruism in viscous populations—an inclusive fitness model. *Evolutionary Ecology 6*, 352–356.

[11] Waldman, B. (1987). Mechanisms of kin recognition. *Journal of Theoretical Biology 128*, 159–185.
